# Viral composition and context in metagenomes from biofilm and suspended growth municipal wastewater treatment plants

**DOI:** 10.1111/1751-7915.13464

**Published:** 2019-08-14

**Authors:** Morgan L. Petrovich, Sarah Ben Maamar, Erica M. Hartmann, Brian T. Murphy, Rachel S. Poretsky, George F. Wells

**Affiliations:** ^1^ Department of Civil and Environmental Engineering Northwestern University 2145 Sheridan Rd., Tech A236 Evanston IL 60208 USA; ^2^ Department of Medicinal Chemistry and Pharmacognosy University of Illinois at Chicago 900 S. Ashland Ave, MBRB Room 3120; MC 870 Chicago IL 60607 USA; ^3^ Department of Biological Sciences University of Illinois at Chicago 950 S. Halsted Street, SEL 4100 Chicago IL 60607 USA

## Abstract

Wastewater treatment plants (WWTPs) contain high density and diversity of viruses which can significantly impact microbial communities in aquatic systems. While previous studies have investigated viruses in WWTP samples that have been specifically concentrated for viruses and filtered to exclude bacteria, little is known about viral communities associated with bacterial communities throughout wastewater treatment systems. Additionally, differences in viral composition between attached and suspended growth wastewater treatment bioprocesses are not well characterized. Here, shotgun metagenomics was used to analyse wastewater and biomass from transects through two full‐scale WWTPs for viral composition and associations with bacterial hosts. One WWTP used a suspended growth activated sludge bioreactor and the other used a biofilm reactor (trickling filter). *Myoviridae, Podoviridae* and *Siphoviridae* were the dominant viral families throughout both WWTPs, which are all from the order *Caudovirales*. Beta diversity analysis of viral sequences showed that samples clustered significantly both by plant and by specific sampling location. For each WWTP, the overall bacterial community structure was significantly different than community structure of bacterial taxa associated with viral sequences. These findings highlight viral community composition in transects through different WWTPs and provide context for dsDNA viral sequences in bacterial communities from these systems.

## Introduction

Viruses are known to be highly abundant throughout wastewater treatment plants (WWTPs), with approximately 10^8^ to 10^10^ virus‐like particles per ml in wastewater and activated sludge (Otawa *et al*., 2007; Rosario *et al*., 2009; Wu and Liu, 2009; Tamaki *et al*., 2012). Wastewater influent and effluent, activated sludge, and reclaimed water derived from wastewater effluent can contain significantly higher viral loads than those found in drinking water or water from natural freshwater bodies (Rosario *et al*., 2009; Tamaki *et al*., 2012). Viruses are relevant to water quality and are concerning in WWTPs because certain viruses are pathogenic and have negative human health impacts, such as hepatitis and gastroenteritis, as a result of exposure (Nordgren *et al*., 2009; Prado *et al*., 2011; Bibby and Peccia, 2013). In addition, viral communities in aquatic systems, including in wastewater treatment infrastructure, include large numbers of bacteriophage (phage) specific to bacterial hosts that are thought to strongly influence bacterial community structure and dynamics (Weinbauer and Rassoulzadegan, 2004). Phage can also pose risks to human health by facilitating horizontal gene transfer of harmful genes and by enhancing pathogenicity of bacteria (Fortier and Sekulovic, 2013; Lood *et al*., 2017).

While several studies have analysed viromes of wastewater, these studies have focused on samples that have been filtered to exclude bacteria in the sampling and DNA extraction process (Wu and Liu, 2009; Tamaki *et al*., 2012; Aw *et al*., 2014), and therefore exclude many viruses such as prophages that are contained within bacteria. In addition, assessment of the virome alone in wastewater treatment infrastructure makes it difficult to directly assess associations with and potential influences on the co‐resident bacterial communities. Furthermore, very little is known about differences (if any) in viral composition between common wastewater treatment process variations, particularly between systems that use suspended versus attached bacterial growth for secondary treatment. Differences could result from the fact that WWTPs using biofilm and suspended growth forms of secondary treatment can contain microbial communities that collectively achieve similar outcomes for nutrient removal, but relative abundances of specific bacterial taxa tend to vary across different types of WWTPs (Wagner and Loy, 2002; Jo *et al*., 2016). Some viruses correspond to specific host taxa, thus the bacterial community composition may influence viral community structure, and simultaneously, viruses can influence bacterial communities by causing bacterial mortality (Fuhrman and Schwalbach, 2003).

Phage infect bacterial cells either as part of the lytic cycle or as prophages in the lysogenic cycle (Desiere *et al*., 2001; Guttman *et al*., 2005; Fortier and Sekulovic, 2013). Lytic phages insert their genetic material into bacteria and replicate into new virions, then eventually lyse the encapsulating bacterial cell (Fortier and Sekulovic, 2013). Prophages, on the other hand, are integrated into the chromosomal DNA of their bacterial host as part of a lysogenic infection process and can be later triggered to re‐emerge as lytic phages when the host is exposed to specific stressors (Guttman *et al*., 2005; Fortier and Sekulovic, 2013). Both lytic and lysogenic phages play important roles in the microbial communities of WWTPs and other aquatic environments. Lytic phages have significant influence on bacteria by killing cells as part of the viral replication process and by transferring genetic material, while prophages can shape communities by enhancing pathogenicity and driving bacterial evolution (Desiere *et al*., 2001; Figueroa‐Bossi *et al*., 2001; Canchaya *et al*., 2004; Fortier and Sekulovic, 2013; Matos *et al*., 2013; Lood *et al*., 2017). Furthermore, prophages play an important role in horizontal transfer of genetic material, including pathogenicity islands and antibiotic resistance genes (ARGs), through transduction (Zhang and LeJeune, 2008; Chen and Novick, 2009; Lood *et al*., 2017). Prophage content in bacterial chromosomes can provide selective advantages for pathogenic bacteria including protection against additional phage infection, and prophages can help bacteria survive in competitive environments (Desiere *et al*., 2001; Fortier and Sekulovic, 2013). Lytic phages and prophages are thought to be key drivers of bacterial community composition and dynamics within natural and engineered aquatic systems, and bacteria in turn act as hosts that support diverse viral communities in these systems (Weinbauer and Rassoulzadegan, 2004; Fortier and Sekulovic, 2013). While bacterial hosts to viruses have been analysed in raw sewage (Cantalupo *et al*., 2011), bacteria associated with lytic and lysogenic phages have not been well characterized across transects of different types of WWTPs.

In this study, we employed high‐throughput metagenomic sequencing to characterize dsDNA viral and bacterial community composition and diversity in wastewater and biomass samples from transects throughout two full‐scale WWTPs that implement different types of secondary treatment processes. One WWTP uses a suspended microbial growth (activated sludge) bioprocess and the other uses an attached growth (trickling filter) bioprocess for secondary treatment. Our results showed that similar families of viral sequences were present throughout the two WWTPs but in different relative abundances, and beta diversity analysis revealed that each WWTP as well as each sampling location within each was significantly distinct in terms of viral community composition. Additionally, bacterial community structure of viral hosts was significantly different than the overall bacterial community. In contrast to existing research into viromes that commonly exclude bacteria, our sampling and analysis approach allowed assessment of prophages in addition to assessment of virus–bacteria host associations. This approach also provides insight into similarities between viral and bacterial communities and interactions in suspended growth and biofilm‐based WWTPs.

## Results

### Viruses in metagenomes from Full‐Scale WWTPs

**Figure 1:**
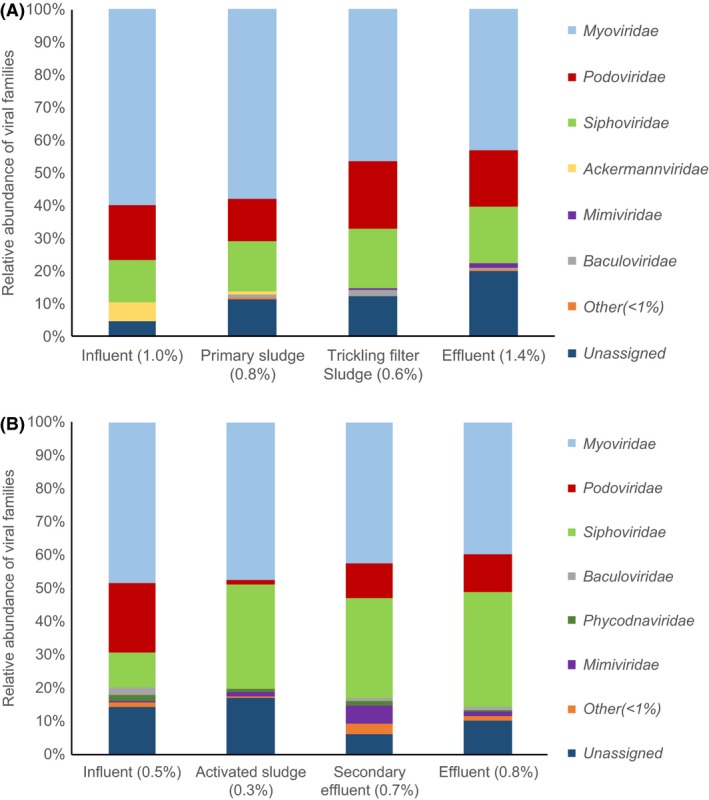
Relative abundance of viral families in WWTPs. Relative abundances are expressed as proportion of total contigs containing viral sequences corresponding to each family based on contig coverage. Percentages next to sample names refer to percent of total contigs that contain viral sequences based on contig coverage. A) Biofilm system. B) Suspended growth system.

A diverse assortment of dsDNA viral sequences was identified in metagenomes from both WWTPs (Fig. 1). When weighted by contig coverage, contigs containing viral sequences comprised between 0.3% and 1.4% of total contigs in the samples. Although the samples were collected using techniques aimed towards bulk biomass capture rather than sampling specifically for only viruses, viral DNA was present in all sampling locations in both WWTPs. Water samples from influent, secondary effluent and final effluent were collected via filtration (see methods for details), while biomass and sludge (activated sludge, primary sludge and trickling filter clarifier sludge) were directly collected for DNA extraction. For water samples, this sampling approach therefore included both lytic and lysogenic phages that are contained within bacteria, as well as any extracellular viruses that adhered to filters during sampling rather than passing through membrane pores. In biomass samples, loss of viruses through membrane pores did not take place, as DNA was extracted directly from biomass without filtering.

Contig assembly statistics are included in Table S1. Average number of contigs and N50 were very similar for the two WWTPs. Despite the difference between water (influent, secondary effluent and final effluent) and biomass or sludge (activated sludge, primary sludge and trickling filter clarifier sludge) sampling approaches, water samples exhibited a higher percentage of contigs containing viral sequences than biomass samples did within each WWTP. In both WWTPs, effluent samples had significantly higher percentages of contigs containing viral sequences than influent samples (Student's *t*‐test, *P *<* *0.05). Overall, the biofilm system contained significantly higher relative abundances of viral sequences than the suspended growth system (Student's *t*‐test, *P *<* *0.05). In the biofilm system, contigs containing viral sequences made up 1.0 ± 0.06% (standard error) of contigs in influent, 0.8 ± 0.06% in primary sludge, 0.6 ± 0.05% in trickling filter clarifier sludge and 1.4 ± 0.15% in effluent. In the suspended growth system, contigs containing viral sequences accounted for 0.5 ± 0.06% of total contigs in influent, 0.3 ± 0.002% in activated sludge, 0.7 ± 0.07% in secondary effluent and 0.8 ± 0.11% in effluent. While types of secondary treatment bioprocesses may drive viral communities in wastewater treatment systems, these differences in viral loading from influent likely also influence viral content throughout the two WWTPs. Contigs that yielded positive BLAST hits to the NCBI Viral RefSeq database for viral sequences were analysed to determine if the length of the aligned portion of the contig represented < 90% of the total contig length, which indicated that the contig did not entirely consist of the assigned viral sequences. In the biofilm system, the percentages of contigs normalized to contig coverage that yielded positive BLAST hits for viral sequences and contained other DNA in addition to viral DNA were 84.5 ± 2.6%, 85.0 ± 2.2%, 93.0 ± 2.2% and 94.9 ± 0.6% (standard error) in the influent, primary sludge, trickling filter clarifier sludge and effluent samples respectively. In the suspended growth system, these percentages were 81.3% ± 1.0%, 89.4% ± 4.1%, 99.1 ± 0.2% and 99.1 ± 0.4% for influent, activated sludge, secondary effluent and effluent respectively. Thus, a large majority of the contigs that were found to contain viral sequences also contained additional DNA that did not align with the viral database in every sampling location in both WWTPs.

### Taxonomic composition of viruses in wastewater and biomass sequences

The vast majority (over 70% in all sampling locations) of viral sequences identified in both the biofilm and suspended growth WWTPs were affiliated with the order *Caudovirales*, which contains the families *Myoviridae, Podoviridae* and *Siphoviridae*. *Caudovirales* relative abundance was slightly higher overall in the biofilm system than the suspended growth system, particularly in the influent (91.2% in the biofilm system versus 70.2% in the suspended growth system). However, this was not the case for the effluent where the suspended growth system had a higher relative abundance of *Caudovirales* than the biofilm system (85.9% in the suspended growth system compared to 71.6% in the biofilm system). When the biofilm and suspended growth WWTPs in this study were screened for the known human pathogenic viruses adenovirus, herpesvirus, papillomavirus and poxvirus, only herpesvirus was detected, and the strains that were identified were not specific to humans.

The overall breakdown of taxonomy of viral sequences (based on alignment with the NCBI RefSeq Viral Database) at the family level is shown in Fig. 1. In both the biofilm system (Fig. 1A) and the suspended growth system (Fig. 1B), viral sequences were predominantly affiliated with the family *Myoviridae* (order: *Caudovirales*). In the biofilm system, *Podoviridae* and *Siphoviridae* comprised similar proportions of viral sequences in all sampling locations, whereas in the suspended growth system, *Siphoviridae* was more abundant than *Podoviridae* in all samples except for the influent. The only other two families that comprised over 5% of viral sequences in any given sampling location were *Ackermannviridae* in the influent to the biofilm system (5.6% ± 1.3%, standard error) and *Mimiviridae* in secondary effluent from the suspended growth system (5.4% ± 1.5%). In the suspended growth system, *Siphoviridae* made up a similar percentage of viral sequences in activated sludge (31.6% ± 6.5%) and effluent (34.7% ± 8.4%). *Myoviridae* relative abundance was somewhat similar (47.4% ± 3.4% and 39.6% ± 7.3% in activated sludge and effluent respectively), but *Podoviridae* was much lower relative abundance in activated sludge (1.4% ± 0.2%) than in effluent (11.6% ± 2.5%).

### Viral diversity throughout attached and suspended growth WWTPs

When diversity of assigned viral sequences in contigs was analysed based on number of unique viral species (species richness), the diversity was higher in the effluent than in the influent in both WWTPs (Fig. S1). In the biofilm system (Fig. S1A), 266 ± 49 (standard error) unique viral species were identified in the effluent compared to 202 ± 27 in the influent, and in the suspended growth system (Fig. S1B), 176 ± 36 unique viral species were identified in the effluent versus 80 ± 24 in the influent. In the biofilm system, both the influent and effluent exhibited greater viral species diversity than biomass samples from the primary and trickling filter clarifier sludge. However, these differences were not statistically significant (Student's *t*‐test, *P *>* *0.05). In the suspended growth system, secondary effluent exhibited the greatest species diversity of viral sequences of any sampling location from the WWTP, with 255 ± 13 unique viral species (Student's *t*‐test, *P *<* *0.05). When alpha diversity was compared via the Shannon Index rather than species richness, viral diversity was slightly higher in the effluent than in the influent for each WWTP, but this was also not statistically significant (Student's *t*‐test, *P *>* *0.05) (Fig. S2). Trends in the suspended growth WWTP based on the Shannon Index were similar to the diversity based on unique species, as secondary effluent had significantly higher diversity than influent and activated sludge (Student's *t*‐test, *P *<* *0.05).

**Figure 2:**
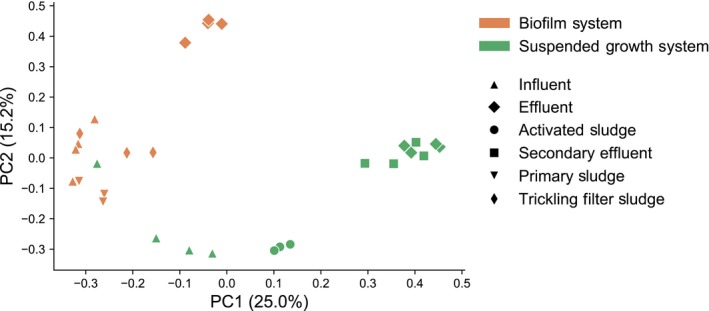
Principal coordinates analysis (PCoA) summarizing beta diversity of viral community composition between sampling locations and WWTPs based on a Bray–Curtis distance matrix at the species level.

A principal coordinates analysis (PCoA) plot summarizing beta diversity of viral communities in the WWTP samples based on the Bray–Curtis distance metric is shown in Fig. 2. Viral communities clustered significantly both by individual WWTP (PERMANOVA pseudo‐*F* test statistic = 6.15, *P *<* *0.01) and by specific sampling location (eight total sampling locations, four per plant, PERMANOVA pseudo‐*F* test statistic = 8.17, *P *<* *0.01). When specific sampling locations were compared for each plant in separate analyses (i.e. two individual analyses, each including four sampling locations from a single plant), samples from each sampling location also showed significant differences (*P *<* *0.01 for each plant and pseudo‐*F* test statistics of 8.94 and 6.06 for the biofilm and suspended growth systems respectively). Therefore, the two WWTPs exhibited distinct overall viral communities when compared to one another and detected viral sequences also varied significantly within each WWTP across sampling transects.

### Composition of the bacterial community associated with viruses and the overall bacterial community

**Figure 3:**
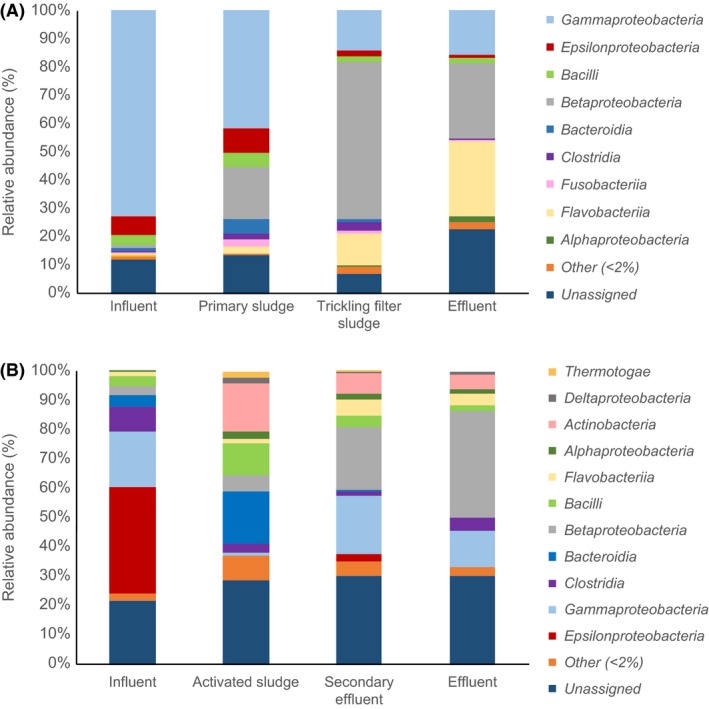
Relative abundance of bacterial classes associated with detected viral sequences. A. Biofilm system. B. Suspended growth system.

When bacterial taxonomy of contigs containing viral sequences was analysed, high variability between WWTPs as well as between samples within a given WWTP was observed (Fig. 3). All contigs containing viral sequences were analysed to determine if those contigs also contained bacterial DNA that could be taxonomically identified in order to identify linkages between viral and bacterial DNA sequences. The percentage of total contigs from each sample (normalized to coverage based on reads mapping) that contained both viral and bacterial DNA in the biofilm system were 0.92 ± 0.07% (standard error) in the influent, 0.74 ± 0.06% in the primary sludge, 0.60 ± 0.04% in the trickling filter sludge and 1.04 ± 0.10% in the effluent. In the suspended growth system, the percentages of total contigs containing both viral and bacterial DNA were 0.38 ± 0.05% in the influent, 0.21 ± 0.01% in the activated sludge, 0.46 ± 0.07% in the secondary effluent and 0.57 ± 0.04% in the final effluent. Bacteria affiliated with the class *Gammaproteobacteria* made up 73.2% of viral hosts in influent to the biofilm system and was also the dominant bacterial class of contigs associated with viral sequences in primary clarifier sludge from this system (41.8%). However, *Gammaproteobacteria* affiliated contigs only made up 19.2% of viral hosts in influent to the suspended growth system. *Gammaproteobacteria* associated with viral sequences decreased throughout the biofilm system, making up only 15.5% of viral hosts in the effluent. In the biofilm system, *Betaproteobacteria* made up the greatest proportion of viral hosts in trickling filter clarifier sludge (55.1%) and was equal in proportion to *Flavobacteriia* in the effluent (26.5% each). Proportions of *Flavobacteriia* associated with viral sequences increased between influent and effluent in the biofilm system. *Epsilonproteobacteria* was the dominant host taxa associated with viral sequences in influent to the suspended growth system (36.2%), particularly taxa affiliated with *Arcobacter tropharium* and the human gastrointestinal pathogen *Arcobacter butzleri* (Arguello *et al*., 2015). However, *Epsilonproteobacteria* only made up 2.6% of viral hosts in the secondary effluent and < 1% in the activated sludge and final effluent samples. In effluent from the suspended growth WWTP, *Betaproteobacteria* had the greatest relative abundance of any class of viral hosts (36.3%) but made up only a small percentage of influent hosts (3.3%).

**Figure 4:**
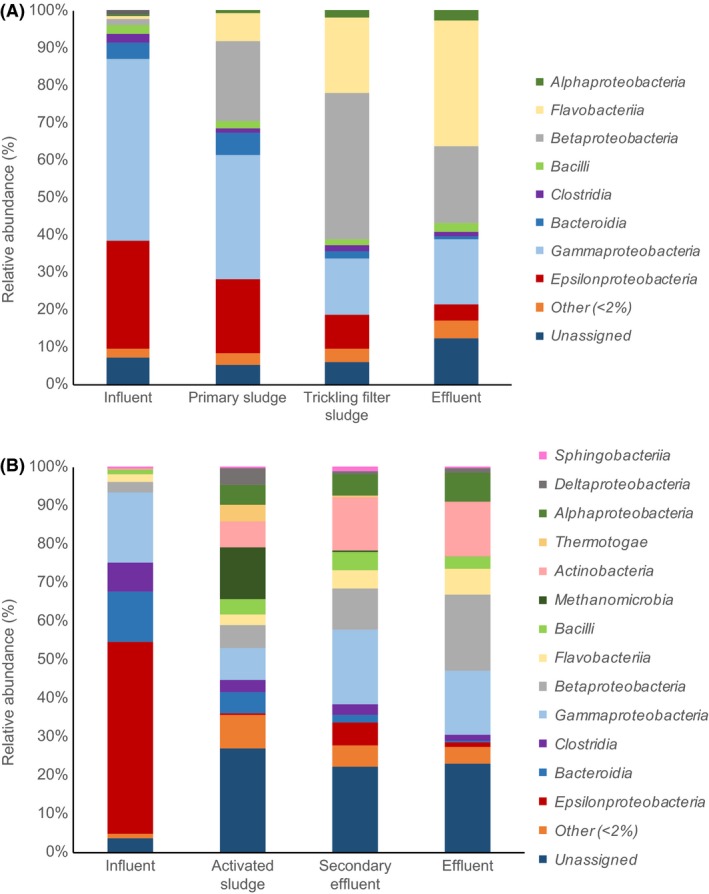
Relative abundance of bacterial classes in the overall bacterial community in the two WWTPs based on taxonomic assignment of contigs. A. Biofilm system. B. Suspended growth system.

**Figure 5:**
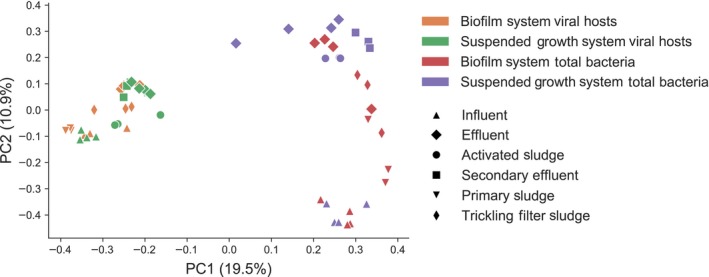
Principal coordinates analysis (PCoA) summarizing community composition beta diversity of bacteria associated with viral sequences and total bacteria based on a Bray–Curtis distance matrix at the species level.

We also employed shotgun metagenome data to analyse the overall bacterial community throughout the two WWTP systems (Fig. 4), in order to compare bacteria hosting viral sequences with overall bacterial community structure. While similarities existed between overall bacterial community composition and composition of bacteria associated with viral sequences in each WWTP, the community structure showed significant differences between the total bacterial community and the taxonomic distribution of bacterial sequences specifically associated with viral sequences on the same contig (PERMANOVA pseudo‐*F* test statistic = 12.8, *P *<* *0.01). This is illustrated in a principal coordinates analysis plot (Fig. 5). For each individual WWTP, similar bacterial classes were observed in each sampling location, but the proportions of these classes varied throughout the systems. In the biofilm system (Fig. 4A), *Gammaproteobacteria* and *Epsilonproteobacteria* were highest in the influent and decreased in relative abundance over the course of treatment, whereas *Betaproteobacteria* and *Flavobacteriia* increased in relative abundance between influent and effluent. In the suspended growth system (Fig. 4B), *Betaproteobacteria* increased in relative abundance of total bacteria over the course of treatment in the suspended growth system, which was also the case for relative abundance of viral hosts. *Gammaproteobacteria* had very similar relative abundances of total bacteria in influent (18.0%) and effluent (16.7%). Interestingly, in the overall bacterial community, *Actinobacteria* increased in relative abundance between activated sludge and final effluent, whereas the relative abundance of *Actinobacteri*a in the subset of the overall community that was identified as a putative viral hosts decreased between these two sampling points. *Epsilonproteobacteria* dominated the overall bacterial community in the influent of the suspended growth system, accounting for 49.8% of all classes, but was not highly represented in any other samples. Less than 10% of any bacterial class was associated with viral sequences, including < 3% for most classes (Fig. S3). In the suspended growth system, proportions of specific bacterial classes that were associated with viral sequences were often higher in effluent than in influent, particularly for *Betaproteobacteria, Clostridia, Deltaproteobacteria, Flavobacteriia* and *Gammaproteobacteria*. This trend was also observed for *Betaproteobacteria, Clostridia* and *Flavobacteriia* in the biofilm system.

## Discussion

### Relative abundances and composition of viruses and hosts

Our results revealed that viral community composition of the two WWTPs was significantly different, yet the same dominant viral families were present throughout both systems. Family‐level similarities reflected findings from previous studies of WWTP viromes in which samples were collected and purified to exclude bacteria (Tamaki *et al*., 2012; Alhamlan *et al*., 2013). This indicates that the same viral families are highly prevalent in many types of WWTPs even when different sampling methods are used, yet finer‐level community structures are distinct for each type of WWTP in this study.

In all sampling locations from the two WWTPs, viral sequences associated with the order *Caudovirales* were dominant and were present in at least 70% of total contigs containing viral sequences. These findings are consistent with previous metagenomics‐based analyses of wastewater (Parsley *et al*., 2010a,2010b; Tamaki *et al*., 2012; Alhamlan *et al*., 2013; Aw *et al*., 2014). The *Caudovirales* order of viruses consists of tailed bacteriophages (Ackermann, 1998). Parsley *et al*. used metagenomic sequences to investigate viruses in activated sludge and found that 95% of identifiable sequences from activated sludge samples belonged to the *Caudovirales* order, with the highest proportion of those assigned to the family *Myoviridae* (40.3%) (Parsley *et al*., 2010a,2010b). This is the same trend that we observed in both the suspended growth and biofilm WWTPs (Fig. 1). Tamaki *et al*. analysed concentrated and purified viral samples from a WWTP and found that 93.8% of assigned viral sequences were annotated as *Caudovirales*. They also found that *Myoviridae* was the most common family in the influent to the WWTP, whereas in effluent and activated sludge *Podoviridae* was the most represented family (Tamaki *et al*., 2012). An analysis of untreated sewage as well as a study on dairy lagoon wastewater, which both used purified viral samples, determined that *Siphoviridae* was the dominant family (Alhamlan *et al*., 2013). In the suspended growth system that we examined, *Siphoviridae* was also abundant, and almost as common as *Myoviridae*, particularly in secondary and final effluent samples. The variations in relative abundances of viral taxa between the two WWTPs could be a result of the fact that suspended growth wastewater treatment systems harbour distinct bacterial communities from biofilm systems (Wagner and Loy, 2002; Jo *et al*., 2016), and these different bacterial communities may select for different corresponding viral communities. Relative abundances of bacteria associated with viral sequences (putative viral hosts) varied between influent and effluent for each WWTP. Previous investigations into viral abundance throughout WWTPs have indicated that absolute abundance of viruses (per ml) is greater in influent than in effluent based on SYBR Green staining and epifluorescence microscopy (Wu and Liu, 2009), but these focused on viromes sampled to exclude viruses contained within bacteria. Prior studies have indicated that removal of viruses differs throughout various wastewater treatment processes (Ewert and Paynter, 1980; Wu and Liu, 2009; La Rosa *et al*., 2010).

Since the vast majority of existing research on viruses in wastewater treatment systems has focused on analyses of viral communities alone rather than samples containing both bacterial and viral sequences, it is unclear how associations between viruses and bacteria within these two WWTPs compare with other WWTPs. Additional research on this topic is warranted. Furthermore, while the present study highlights overall associations between bacterial and viral sequences, future research is needed to evaluate associations between bacterial taxa and lysogenic compared to lytic viruses. This in turn would further clarify which bacterial taxa are particularly likely to be associated with prophages and which are most prone to infection by lytic viruses. In addition, we found that specific classes of bacterial contigs associated with viral sequences were enriched in relative abundance over the course of wastewater treatment in the two systems, including contigs affiliated with *Betaproteobacteria* and *Flavobacteriia*, and others such as *Gammaproteobacteria* decreased in relative abundance between influent and effluent. These trends may be due to progressive removal along the course of the treatment of gut‐associated bacteria as numerous representatives are found in the *Gammaproteobacteria* class as well as in the *Epsilonproteobacteria* class (Rizzatti *et al*., 2017; Moon *et al*., 2018), while *Betaproteobacteria* and *Flavobacteriia* class bacteria are frequently encountered in freshwater (Mun *et al*., 2013; Llirós *et al*., 2014) as well as in activated sludge and other secondary treatment bioprocesses (Wells *et al*., 2014; Wu *et al*., 2019). The relative abundance of the viral sequences associated with these different bacteria likely follow the abundance of their host which could explain similar trends between the overall bacterial community composition and the hosts’ community composition. The overall bacterial community composition at each sampling location and WWTP constitutes various potential hosts for free viruses. As the different treatment systems and processes directly influence the bacterial community structure in each treatment plant through their various physico‐chemical conditions (substrate concentration, dissolved oxygen, aggregate structure, etc.), the different treatment processes are also likely to directly and indirectly influence the viral community structures observed at each sampling point. However, while similar qualitative trends were observed in the overall bacterial community, the relative abundances of taxa differed significantly for bacteria associated with viral sequences compared to the overall bacterial community.

### Diversity of viral sequences throughout full‐scale wastewater treatment systems

Wastewater contains one of the most diverse viromes of any environment that has been studied to date, likely related to the dense conditions and high diversity of bacteria that viruses can infect (Cantalupo *et al*., 2011). Activated sludge also contains extremely high concentrations of virus particles compared to other environments and exhibits high diversity (Otawa *et al*., 2007; Wu and Liu, 2009). Cantalupo *et al*. identified 234 known viruses in raw sewage, yet most viral sequences could not be classified, indicating high diversity and highlighting some of the challenges involved in studying viruses using next‐generation sequencing methods (Cantalupo *et al*., 2011). Aw *et al*. also observed that only 11.3% of contigs in viral samples from sewage could be assigned using the NCBI RefSeq database (Aw *et al*., 2014), and 80–95% of sequences from viral samples taken throughout a WWTP by Tamaki *et al*. were unassigned (Tamaki *et al*., 2012). As shown in Fig. 1, we documented a much lower number of unassigned viral sequences (5–20%) at the family level in this study, but that is due to the fact that those viral sequences did positively align against the viral reference database and were assigned at higher taxonomic levels but were taxonomically unassigned at the family level. While reference databases are constantly being expanded, the large fraction of unknown sequences in the WWTP samples illustrates the extent to which viral communities in these environments are still largely uncharacterized and extremely diverse. Since our sampling strategy resulted in a metagenomic dataset that includes both bacterial as well as viral sequences, it is unclear exactly how many viral sequences were unassigned, as any contigs that did not positively align with the virus database likely often corresponded to bacteria that did not contain viral sequences, though some could have potentially contained unidentifiable viral sequences as well.

### Relevance of viruses in natural and engineered aquatic environments

Viruses play an important role in shaping the microbial communities of both natural and engineered environments by killing large numbers of bacterial cells and driving bacterial evolution (Otawa *et al*., 2007; Fortier and Sekulovic, 2013). Suttle (1994) estimated that up to 20% of marine bacteria are infected by viruses. Lytic phages infect bacteria, replicate inside of them, then lyse the cells to release large numbers of new viruses into the environment (Fortier and Sekulovic, 2013). As many as 10‐20% of bacteria in aquatic environments are killed per day as a result of this cycle, which can influence the species composition and diversity of overall communities (Suttle, 1994; Weinbauer and Rassoulzadegan, 2004; Otawa *et al*., 2007). Viruses may infect and lyse dominant bacterial taxa, which gives less competitive species more opportunity to survive, and the destruction of this large proportion of cells releases organic matter into ecosystems which can be consumed by other organisms (Thingstad and Lignell, 1997; Weinbauer and Rassoulzadegan, 2004). In wastewater treatment bioreactors, the presence and activity of lytic viruses could be both detrimental to functional taxa that aid in nutrient or organic carbon removal and beneficial by killing pathogenic microorganisms. Prophages, which incorporate into the chromosomes of bacteria and can later re‐emerge into the lytic form, influence the microbial world contained within aquatic environments in other ways and are likely to constitute a significant portion of the viral community of WWTP samples that include bacteria. For example, prophages can facilitate horizontal gene transfer which can impact diversity and evolution of bacteria (Weinbauer and Rassoulzadegan, 2004; Parsley *et al*., 2010a,2010b; Fortier and Sekulovic, 2013; Lood *et al*., 2017; Stachler *et al*., 2019). Thus, both lytic phages and prophages are important in WWTP viral communities.

## Conclusions

Overall, when viral sequence composition was analysed in the context of bacterial communities in suspended growth and biofilm‐based WWTPs, results indicated that the biofilm system had higher overall relative abundances of viral sequences as a proportion of total sequences than the suspended growth system. This observation could be the result of a higher local density of microorganisms in the biofilm system fostering multiplication of viruses or the trapping of the viruses in the extracellular exopolysaccharide matrix. However, this is not necessarily a direct result of the secondary treatment process differences, as relative proportions of contigs containing viral sequences in the influent were also higher in the biofilm system than in the suspended growth system. Still, higher relative abundances of viruses in certain wastewater streams may indicate that viruses have greater potential to influence bacterial community composition and facilitate virus‐mediated horizontal gene transfer. In both WWTPs, bacteriophages from the order *Caudovirales* made up a majority of identifiable viral sequences, which is consistent with previous findings. Viral community structure at the family level was similar at different sampling locations within each plant as well between the two plants, yet taxonomy of bacteria associated with these viral sequences showed high variation across different sampling sites and across the two WWTPs, potentially reflecting the broad bacterial host range of certain viruses. Significant differences were found between community structure of bacteria associated with viral sequences and total bacterial communities.

Our findings elucidate the spatial dynamics of viruses within metagenomes obtained from transects through two WWTPs, sampled so as to include both viral dsDNA and bacterial DNA. This in turn provides context within the bacterial community for the presence of these viruses. In future research, samples collected using methods to include both bacterial and viral content should be compared to samples from the same location specifically concentrated and purified for viral content in order to identify similarities and differences between their viral populations. In addition, stressors such as UV disinfection and presence of trace pharmaceuticals such as antibiotics in wastewater treatment systems can activate prophages to change to the lytic cycle (Fortier and Sekulovic, 2013), and so future work is warranted to understand how specific treatment processes and wastewater compositions can influence relationships between viruses and bacteria.

## Experimental procedures

### Sampling and sequencing methods

Samples were collected from two full‐scale WWTPs in Wisconsin as described previously (Petrovich *et al*., 2018). Briefly, the Sheboygan Regional WWTP contains a suspended growth activated sludge bioreactor, and the Manitowoc WWTP contains a trickling filter (biofilm reactor). The two WWTPs serve similarly sized populations in towns located about 30 miles apart, and secondary treatment systems were implemented in both WWTPs in the late 1950s. Influent to each system consists of approximately 75% municipal and 25% industrial wastewater. Sampling locations from the Sheboygan Regional WWTP included primary effluent (four samples), activated sludge from the aeration basin (three samples), secondary effluent (four samples) and final effluent (four samples). At the Manitowoc WWTP, sampling locations included primary effluent (four samples); sludge from the primary clarifier, referred to as ‘primary sludge’ (three samples); secondary sludge taken from the clarifier that immediately follows the trickling filters, considered to be a proxy for microbial biomass in the trickling filter and referred to as ‘trickling filter clarifier sludge’ (three samples); and final effluent (four samples). Water samples were initially filtered through a 1.6 μm pore size glass microfibre Whatman filter (GE Healthcare, Amersham, UK), then were filtered through 0.22 μm pore size Sterivex filters (EMD Millipore, Darmstadt, Germany). Genomic DNA was extracted from water filters using phenol–chloroform DNA extraction methods as previously described (Oh *et al*., 2011). PowerSoil DNA Isolation Kits (MoBio Laboratories, Carlsbad, CA, USA) were used to extract genomic DNA from thoroughly mixed biomass samples per the manufacturer's instructions. Shotgun metagenomics sequencing methods were previously described (Petrovich *et al*., 2018) and are included in the Supporting Information. Sequences are available through NCBI under the SRA accession number SRP107015.

### Assembly of metagenomic sequences

Raw reads were checked for quality using FastQC, then trimming was performed with CutAdapt (Martin, 2011). The maximum allowed error rate (−e) was set to 0.1, quality (−q) = 20, overlap (−O) = 5 and reads shorter than 20 were discarded (−m). IDBA‐UD (Peng *et al*., 2012) was then used to assemble clean reads into contigs using the options −mink 21 −maxk 101 −step 10 −min_contig 500 −num_threads 28. Assemblies were performed for each sample replicate separately. Sequencing reads from each sample were mapped to contigs, and of contigs was calculated with Bowtie2 (Langmead and Salzberg, 2012) and SAMtools v1.2 (Li *et al*., 2009).

### Identification of viruses, diversity and associations with bacterial taxa

Contigs were aligned against the NCBI RefSeq Viral Database (downloaded August 2018) (O'Leary *et al*., 2015) with BLASTn using the cut‐off *e*‐value ≤ 10^−5^ (Tamaki *et al*., 2012; Kim *et al*., 2017). Taxonomy of viral sequences was determined based on taxonomic IDs of viral BLAST hits. This approach was selected because it provides a link between viral DNA in contigs and bacterial markers contained within those contigs and allows comparison of viral and bacterial ecology between samples. Thus, it was possible to identify viral DNA and simultaneously identify taxonomy of bacteria for contigs containing both viral DNA and bacterial markers. While a variety of methods are available for identifying and annotating viruses in metagenomes, the advantage of the approach used here is that it provided a way of combining viral and bacterial analyses for individual contigs.

At the order level, percentage of viral sequences in samples associated with the *Caudovirales* was determined by dividing read coverage of contigs containing sequences assigned to *Caudovirales* by total coverage of all contigs containing viral sequences. Taxonomic analysis of viral sequences at the family level was performed by taking best hits for each viral taxonomic ID per contig, summing coverages of these contigs containing viral sequences for each family, then dividing the total contig coverage of each family by the total coverage of contigs containing viral sequences for that sample. Viral alpha diversity was calculated as number of unique viral species (richness) in each sample as well as by the Shannon diversity index, based on viral sequences that could be annotated to the species level. Statistically significant differences between alpha diversity of viral sequences in various sampling locations were determined with Student's *t*‐tests calculated in Excel v.15.27. Beta diversity was analysed using the Scikit‐Bio Python package (http://scikit-bio.org/) using a Bray–Curtis distance matrix based on taxonomic content of each sample at the species level for both viruses and bacteria as a result of BLAST alignment against the NCBI RefSeq Viral database. PERMANOVA statistical analyses comparing beta diversity between WWTPs and across different sampling locations were also calculated with Scikit‐Bio. Principal coordinates analysis plots were generated using the Matplotlib (https://matplotlib.org/) and Seaborn (https://seaborn.pydata.org/) Python packages. Samples were screened for specific DNA viruses that are known to be pathogenic to humans, including adenovirus, herpesvirus, papillomavirus and poxvirus based on taxonomic assignment of hits from the NCBI RefSeq Viral database.

In order to infer potential bacterial hosts for observed viruses, contigs containing viral sequences were analysed with Kraken2 software using default parameters (Wood and Salzberg, 2014) to determine if bacterial DNA was also present on those contigs that could be taxonomically assigned. Taxonomy was assigned by Kraken to all contigs that contained both bacterial markers and putative prophage DNA sequences based on alignment against the NCBI Viral RefSeq database with a cut‐off *e*‐value ≤ 10^−5^. The resolution to which taxonomy could be assigned varied by contig, and taxonomy was assigned to the species level where possible. In addition to contigs containing viral sequences, all contigs were also analysed with Kraken2 in order to characterize bacterial community composition of any contigs containing bacterial DNA.

## Conflict of interest

None declared.

## Supporting information


**Fig. S1.** Diversity of viruses in WWTPs, based on species richness (unique species). Error bars represent standard error of sample replicates. Percentages refer to percent of total contigs that contain viral sequences, based on coverage. (A) Biofilm system. (B) Suspended growth system. In the biofilm system, differences in diversity between sampling sites were not significant (Student's *T*‐Test, *P *> 0.05). In the suspended growth system, secondary effluent had significantly higher diversity than all other sampling locations (Student's *T*‐Test, *P* < 0.05).
**Fig. S2.** Alpha diversity of viruses in WWTPs, based on the Shannon diversity index. Error bars represent standard error of sample replicates. (A) Biofilm system. (B) Suspended growth system. In the biofilm system, differences in diversity between sampling sites were not significant (Student's *T*‐Test, *P* > 0.05). In the suspended growth system, secondary effluent had significantly higher diversity than influent and activated sludge, and diversity in effluent was significantly higher than in influent (Student's *T*‐Test, *P* < 0.05).
**Fig. S3.** Percentage of total bacterial contigs with class‐level taxonomic affiliation that were associated with viruses. (A) Biofilm system. (B) Suspended growth system.
**Table S1.** Contig assembly statistics for samples from the biofilm system and suspended growth system.
