# Supplementary material for: Viral composition and context in metagenomes from biofilm and suspended growth municipal wastewater treatment plants
Source: Microb Biotechnol. 2019 Aug 14;12(6):1324–36. doi: 10.1111/1751-7915.13464 (PMC6801142; doi:10.1111/1751-7915.13464)
Supplement: Supplementary file 1 — Fig. S1. Diversity of viruses in WWTPs, based on species richness (unique species). Error bars represent standard error of sample replicates. Percentages refer to percent of total contigs that contain viral sequences, based on coverage. (A) Biofilm system. (B) Suspended growth system. In the biofilm system, differences in diversity between sampling sites were not significant (Student's T‐Test, P > 0.05). In the suspended growth system, secondary effluent had significantly higher diversity than all other sampling locations (Student's T‐Test, P < 0.05). Fig. S2. Alpha diversity of viruses in WWTPs, based on the Shannon diversity index. Error bars represent standard error of sample replicates. (A) Biofilm system. (B) Suspended growth system. In the biofilm system, differences in diversity between sampling sites were not significant (Student's T‐Test, P > 0.05). In the suspended growth system, secondary effluent had significantly higher diversity than influent and activated sludge, and diversity in effluent was significantly higher than in influent (Student's T‐Test, P < 0.05). Fig. S3. Percentage of total bacterial contigs with class‐level taxonomic affiliation that were associated with viruses. (A) Biofilm system. (B) Suspended growth system. Table S1. Contig assembly statistics for samples from the biofilm system and suspended growth system. [file MBT2-12-1324-s001.docx]

**Supporting Information**

| **Sample** | **Number of Contigs** | **Largest Contig Size (bp)** | **N50 (bp)** | **Coupled Reads Size (Gbp)** |
| --- | --- | --- | --- | --- |
|  |  |  |  |  |
| **Biofilm System** |  |  |  |  |
| Influent 1 | 35,200 | 169,157 | 3,211 | 3.28 |
| Influent 2 | 36,022 | 217,212 | 3,126 | 3.35 |
| Influent 3 | 23,058 | 166,180 | 3,296 | 1.82 |
| Influent Prefilter | 54,894 | 280,865 | 2,390 | 5.49 |
| Primary Sludge 1 | 55,267 | 93,082 | 2,021 | 3.56 |
| Primary Sludge 2 | 28,141 | 91,876 | 1,794 | 2.16 |
| Primary Sludge 3 | 48,083 | 84,121 | 2,009 | 2.89 |
| Trickling Filter Sludge 1 | 49,023 | 54,230 | 1,763 | 2.92 |
| Trickling Filter Sludge 2 | 87,050 | 73,449 | 1,894 | 5.28 |
| Trickling Filter Sludge 3 | 38,833 | 58,214 | 1,611 | 2.54 |
| Effluent 1 | 29,535 | 267,374 | 3,710 | 2.99 |
| Effluent 2 | 15,843 | 230,910 | 4,286 | 1.95 |
| Effluent 3 | 19,749 | 230,911 | 3,615 | 2.23 |
| Effluent Prefilter | 80,698 | 325,315 | 2,969 | 7.02 |
| *Average* | *42,957* | *167,350* | *2,693* | *3.39* |
|  |  |  |  |  |
| **Suspended Growth System** |  |  |  |  |
| Influent 1 | 30,155 | 119,307 | 2,065 | 2.41 |
| Influent 2 | 39,715 | 99,775 | 1,982 | 2.95 |
| Influent 3 | 13,226 | 56,141 | 2,264 | 1.08 |
| Influent Prefilter | 48,229 | 82,580 | 2,255 | 3.44 |
| Activated Sludge 1 | 31,861 | 407,747 | 2,107 | 2.92 |
| Activated Sludge 2 | 66,164 | 356,990 | 2,649 | 3.05 |
| Activated Sludge 3 | 66,347 | 409,672 | 2,718 | 3.11 |
| Secondary Effluent 1 | 52,671 | 211,689 | 2,824 | 2.98 |
| Secondary Effluent 2 | 62,476 | 211,689 | 3,422 | 3.32 |
| Secondary Effluent 3 | 62,146 | 275,233 | 3,418 | 3.32 |
| Secondary Effluent Prefilter | 67,460 | 223,144 | 1,813 | 3.69 |
| Effluent 1 | 16,655 | 177,685 | 2,785 | 2.31 |
| Effluent 2 | 12,533 | 153,603 | 2,002 | 1.05 |
| Effluent 3 | 47,252 | 269,048 | 2,750 | 2.87 |
| Effluent Prefilter | 32,886 | 223,144 | 2,797 | 2.45 |
| *Average* | *43,318* | *218,496* | *2,523* | *2.73* |

**Table S1:** Contig assembly statistics for samples from the biofilm system and suspended growth system.

A)

B)

**Figure S1:** Diversity of viruses in WWTPs, based on species richness (unique species). Error bars represent standard error of sample replicates. Percentages refer to percent of total contigs that contain viral sequences, based on coverage. **A)** Biofilm system. **B)** Suspended growth system. In the biofilm system, differences in diversity between sampling sites were not significant (Student’s T-Test, p > 0.05). In the suspended growth system, secondary effluent had significantly higher diversity than all other sampling locations (Student’s T-Test, p < 0.05).

A)

B)

**Figure S2:** Alpha diversity of viruses in WWTPs, based on the Shannon diversity index. Error bars represent standard error of sample replicates. **A)** Biofilm system. **B)** Suspended growth system. In the biofilm system, differences in diversity between sampling sites were not significant (Student’s T-Test, p > 0.05). In the suspended growth system, secondary effluent had significantly higher diversity than influent and activated sludge, and diversity in effluent was significantly higher than in influent (Student’s T-Test, p < 0.05).

A)

B)

**Figure S3:** Percentage of total bacterial contigs with class-level taxonomic affiliation that were associated with viruses. **A)** Biofilm system. **B)** Suspended growth system.

**Metagenomics Sequencing Methods**

Libraries were prepared with NeoPrep library preparation (Illumina, San Diego, CA) and DNA was sheared with acoustic shearing (Covaris, MA) to 2x100 base pairs at the University of Illinois at Chicago. Samples were then subjected to shotgun metagenomics sequencing using an Illumina HiSeq2500 (Illumina, San Diego, CA) at the University of Illinois at Urbana-Champaign.
